# Characterization of the seven-day course of pulmonary response following unilateral lung acid injury in rats

**DOI:** 10.1371/journal.pone.0198440

**Published:** 2018-06-04

**Authors:** Florian Setzer, Barbara Schmidt, Lars Hueter, Konrad Schwarzkopf, Jörg Sänger, Torsten Schreiber

**Affiliations:** 1 Department of Intensive Care Medicine, Inselspital, Bern University Hospital, University of Bern, Bern, Switzerland; 2 Department of Anesthesiology and Intensive Care Medicine, Jena University Hospital, Jena, Germany; 3 Department of Anesthesia and Intensive Care, Zentralklinik Bad Berka, Bad Berka, Germany; 4 Department of Anesthesia and Intensive Care, Klinikum Saarbrücken, Winterberg, Saarbrücken, Germany; 5 Laboratory for Pathology and Cytology, Zentralklinik Bad Berka, Bad Berka, Germany; Emory University School of Medicine, UNITED STATES

## Abstract

**Background:**

Aspiration of gastric acid is an important cause of acute lung injury. The time course of the pulmonary response to such an insult beyond the initial 48 hours is incompletely characterized. The purpose of this study was to comprehensively describe the pulmonary effects of focal lung acid injury over a seven day period in both directly injured and not directly injured lung tissue.

**Methods:**

Male Wistar rats underwent left-endobronchial instillation with hydrochloric acid and were sacrificed at 4, 24, 48, 96 or 168 h after the insult. Healthy non-injured animals served as controls. We assessed inflammatory cell counts and cytokine levels in right and left lung lavage fluid and blood, arterial oxygen tension, alterations in lung histology, lung wet-to-dry weight ratio and differential lung perfusion.

**Results:**

Lung acid instillation induced an early strong inflammatory response in the directly affected lung, peaking at 4–24 hours, with only partial resolution after 7 days. A less severe response with complete resolution after 4 days was seen in the opposite lung. Alveolar cytokine levels, with exception of IL-6, only partially reflected the localization of lung injury and the time course of the functional and histologic alterations. Alveolar leucocyte subpopulations exhibited different time courses in the acid injured lung with persistent elevation of alveolar lymphocytes and macrophages. After acid instillation there was an early transient decrease in arterial oxygen tension and lung perfusion was preferentially distributed to the non-injured lung.

**Conclusion:**

These findings provide a basis for further research in the field of lung acid injury and for studies exploring effects of mechanical ventilation on injured lungs. Incomplete recovery in the directly injured lung 7 days after acid instillation suggests that increased vulnerability and susceptibility to further noxious stimuli are still present at that time.

## Introduction

Aspiration of gastric acid, potentially resulting in pneumonitis, is an important cause of acute lung injury and respiratory failure [1, 2] and a potential complication of clinical conditions associated with impaired consciousness due to a variety of reasons, including general anesthesia [3–5].

Pulmonary instillation of hydrochloric acid is used as an experimental model to explore this type of lung injury [6–8] and in addition has been established as a first-hit insult to investigate the effects of mechanical ventilation (= second hit) on injured lungs [9–11].

The immediate and early pulmonary inflammatory response following lung acid injury has been well characterized in animal experiments [12–14]. In contrast, the pulmonary response beyond the first 24–48 hours after such an insult is not as comprehensively investigated and has only recently been addressed in few experimental studies [15–17].

Detailed knowledge of the time course of evolution and resolution of the pulmonary response to a specific insult (such as experimental acid injury) could improve our understanding of how and when injured lungs respond to or may be negatively affected by treatment strategies (e.g. mechanical ventilation to treat respiratory failure caused by acid pneumonitis). In addition, by providing reference data, such knowledge may facilitate planning future experiments employing two-hit models.

In the clinical setting, acute lung injury frequently is patchily distributed with severely injured areas interspaced with less affected tissue [18–20]. Following focal experimental lung acid injury, less pronounced inflammation was shown in distant, not directly injured lung areas [21–23]. Hence ideally, an experimental model used to characterize the time course of the response to such damage should replicate inhomogeneity of tissue integrity and enable investigators to differentiate effects seen in severely affected lung tissue from those evolving in not (or only mildly) affected areas.

Accordingly, we submitted rats to unilateral pulmonary acid instillation and assessed at pre-defined time points over one week the response to this injury. Limiting the insult to one lung only ensured oxygenation and survival during the observation period and offered the opportunity to sample acid instilled and not instilled lung areas separately. To comprehensively characterize the response, we assessed arterial oxygenation, pulmonary and systemic inflammation and lung edema, perfusion and histology.

## Materials and methods

This study was approved by the institutional and local Animal Protection Committee (Thüringer Landesamt für Lebensmittelsicherheit und Verbraucherschutz, Bad Langensalza, Germany; 02-27/04 and 02-011/06). Male Wistar rats from an in-house outbred strain, 10–12 weeks old, weighing 381 (±2) g were used. Rats were housed in the animal facility of the university under standard conditions, at constant temperature (21°C) and a regular day-night cycle with food and water ad libitum. Animals were handled according to the institutional guidelines for the care of laboratory animals and the laws of the state of Thuringia, Germany. Invasive procedures were performed under anesthesia with isoflurane as described below. Experiments were carried out on weekdays, in the designated laboratories of the university. Rats were observed and examined daily during the longitudinal experiments. (S1 Text)

### Induction of lung acid injury

Unilateral lung acid injury was induced as previously described [24, 25]. Briefly, laryngoscopy was performed under isoflurane anesthesia with animals spontaneously breathing and the trachea was intubated with a specially designed cannula with a blunt olive shaped tip. The cannula was advanced into the left main bronchus and 0.4 ml 0.1N hydrochloric acid (HCl) instilled into the left lung. Animals were kept in the left lateral position until emergence from anesthesia (approximately 10 minutes). Extensive experience with this procedure has been acquired in previous experiments and it has been shown that acid deposition is confined to the left lung [24, 25]. Volatile anesthetics were used, because they ensure deep anesthesia for inoculation and fast pharmacokinetics.

### Study groups

#### Randomization

Animals were randomized to experimental groups using sealed envelopes (S1 Timeline).

#### Single point experiments

To obtain biochemical and histological markers of lung injury, animals were sacrificed at 4 h, 24 h, 48 h, 96 h or 168 h after unilateral acid instillation (n = 14 at each time point). Ten animals without lung acid injury, but otherwise treated concordantly (i.e. including anesthesia, but no intrapulmonary instillation), were sacrificed to obtain baseline parameters. In each group, left and right lung lavage was performed in 50% of the animals and left and right lung histology and wet-to-dry weight ratio assessed in the other 50%.

#### Longitudinal experiment

Five animals underwent serial assessment of oxygenation, blood leukocyte counts and lung perfusion prior to and at 4, 24, 48, 96 and 168 hours after unilateral lung acid injury. These animals were sacrificed after 168 hours.

To perform post-mortem assessments and sampling, animals were deeply anesthetized and sacrificed by rapidly withdrawing 8–10 ml blood from the inferior vena cava.

#### Single point experiments: Post mortem measurements and sample collection

Median sternotomy was performed, lungs were removed en-bloc from the thorax and inspected for macroscopic effects of acid instillation. Animals were excluded if right or bilateral lung injury was present.

Lung lavage: Left and right lungs were lavaged separately as previously described using 10 ml of phosphate buffered saline for each lung in aliquots of 2.5 ml [25]. Effluents from each side were centrifuged separately at 3000 rpm for 10 minutes and absolute numbers of neutrophils, macrophages and lymphocytes in the resulting cell pellet were counted using a hemocytometer and corrected for the total amount of lavage fluid obtained.

Protein content in lung lavage fluid supernatant was measured using turbidimetry (assay: Roche Diagnostics; analyzer: Hitachi 717).

Cytokine levels in lung lavage fluid supernatant and in blood plasma were assessed by ELISA using commercially available kits specific for rat (IL-6: KRC0062; MIP-2: KRC1022; IL-1β: KRC0011, IL-10: KRC0101, all: BioSource, Solingen, Germany). Kits were used according to manufacturer guidelines. The detection threshold was 8 pg/ml for IL-6, 1 pg/ml for MIP-2, 3 pg/ml for IL-1β and 5 pg/ml for IL-10.

Procollagen-III-peptide (P-III-P), a marker of collagen III synthesis, was assessed by RIA in lung lavage fluid (RIA-3750, DRG Instruments GmbH, Marburg, Germany) to detect increased collagen turnover indicative of lung fibrosis.

Lung histology: A sagittal section (distance from lung hilus approximately 4–5 mm) of the right and left lung (extending from the apex to the basal part of the lung) was fixed in 10% formalin and hematoxylin and eosin (H&E) and Sirius Red (to assess potential fibrosis) stained tissue sections (5 μm) were prepared.

Lung wet-to-dry weight ratio (W/D-ratio): Left and right lungs were weighed separately before and after the tissue sample for histology had been resected. A representative amount of lung tissue (> 50% of whole left or right lung weight) remained, which was weighed again after exsiccation. Then W/D-ratio was calculated.

#### Longitudinal experiments: Assessment of oxygenation, blood leukocyte counts and lung perfusion

In animals allocated to these experiments tail artery blood was collected and color-coded microspheres were injected intravenously under brief inhalational anesthesia (1.5% isoflurane in 100% oxygen) prior to and at 4, 24, 48, 96 and 168 hours after unilateral lung acid injury.

Arterial oxygen tension was determined using a blood gas analyzer (ABL 50, Radiometer, Copenhagen, Denmark).

Total white blood cell count was determined with a hemocytometer. Percentages of neutrophils and lymphocytes were obtained from a cell smear (May-Gruenwald-Giemsa stain; 100 white blood cells counted at a magnification of x500), and absolute neutrophil and lymphocyte numbers calculated according to the total white blood cell count.

The technique to assess lung perfusion has been described in detail elsewhere [26, 27]. Briefly, using different colours, 100.000 microspheres with a diameter of 15μm (Dye Trak, Triton Technology, San Diego, USA) were injected intravenously at each time point. Injections were performed through a tail vein.

Animals were sacrificed after the last microspheres injection, sternotomy was performed and right and left lungs were harvested, weighed, digested (4N concentrated solution of KOH) and processed separately as previously described, to obtain the microspheres from the pulmonary microvasculature and remove the dye from the microspheres [24]. The spectrophotometric absorption of each dye solution was determined at wavelengths of 190–820 nm and the number of microspheres calculated using the specific absorbance value of the different dyes. Percentage of left and right lung perfusion was calculated as the proportion of the microsphere number on the total number of microspheres.

### Statistical analysis

Data are presented as mean ± SEM. Analysis of variance (ANOVA) with Bonferroni post-hoc correction for multiple comparisons was used to assess the effects of side (right versus left lung) and time on markers of lung injury and inflammation. P < 0.05 was considered statistically significant. The statistics software SPSS, Version 20 (IBM SPSS Statistics, Armonk, NY) was used for data analysis.

## Results

Acid instillation caused transient symptoms of illness in varying degree (slightly reduced locomotion, piloerection, and occasionally, tachypnea) for 4–6 hours. Symptoms were mild and resolved spontaneously in all animals. No deaths occurred. Effects of acid instillation were visible as consolidated areas extending to approximately 50% of the surface of deflated left lungs at 4 and 24 h and to approximately 20–30% of the left lung surface at 48 and 96 h after acid instillation. Residual alterations on left lungs were visible in 5 animals at 168 hours. Left lungs of the other 9 animals in this group appeared normal and comparable to the lungs of non-acid instilled animals. Macroscopically, bilateral lung injury was present in 1 animal in the 24 hours group and right lung injury was present in 1 animal in the 48 hours group. Both animals were excluded from data analysis.

### Single point experiments

#### Lung wet-to-dry weight ratio (Fig 1)

W/D-ratio increased significantly in left lungs at 4 and 24 hours after acid instillation and remained unchanged in right lungs.

**Fig 1:**
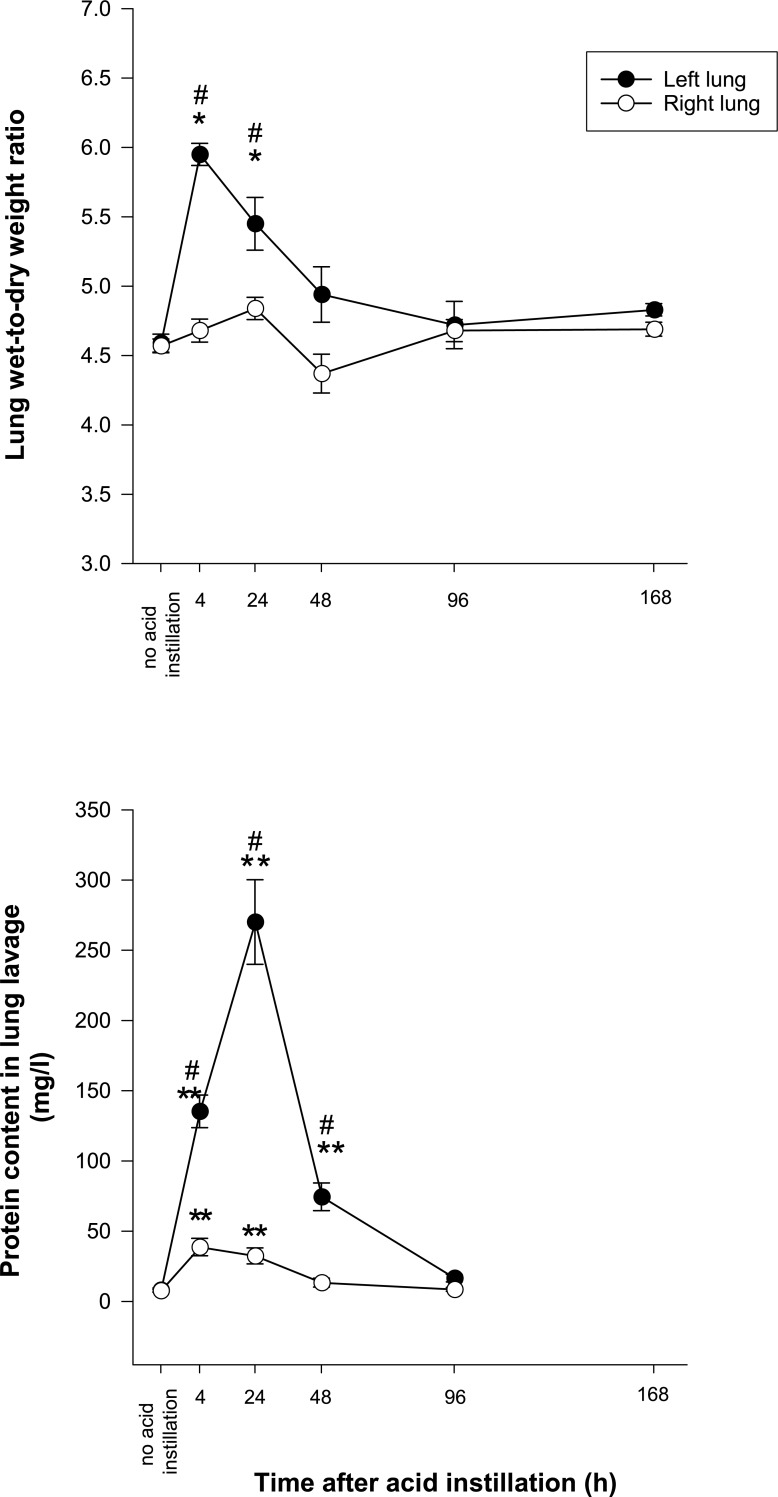
Lung wet-to-dry weight ratio and alveolar protein concentration after left lung acid instillation. * = p < 0.05 vs. no acid instillation. ** = p < 0.001 vs. no acid instillation. # = p < 0.05 vs. right lung.

#### Protein content in lung lavage fluid (Fig 1)

There was a significant increase in lung lavage protein content in both lungs early after acid instillation, but much stronger so in the left lung. Protein concentrations returned to baseline values at 96 h in left and at 48 h in right lungs. For technical reasons and insufficient essay quality, results for the time point 168 h were rejected.

#### Inflammatory cell counts in lung lavage fluid (Fig 2)

Following left lung acid injury there was a significant increase in alveolar leukocyte counts in both lungs, which was characterized by different time courses for leukocyte subpopulations. There was an early peak in alveolar neutrophil counts, which returned to baseline values before the end of the observation period and a delayed increase in alveolar macrophage and lymphocyte counts (with numbers remaining increased in left lungs at 7 days).

**Fig 2:**
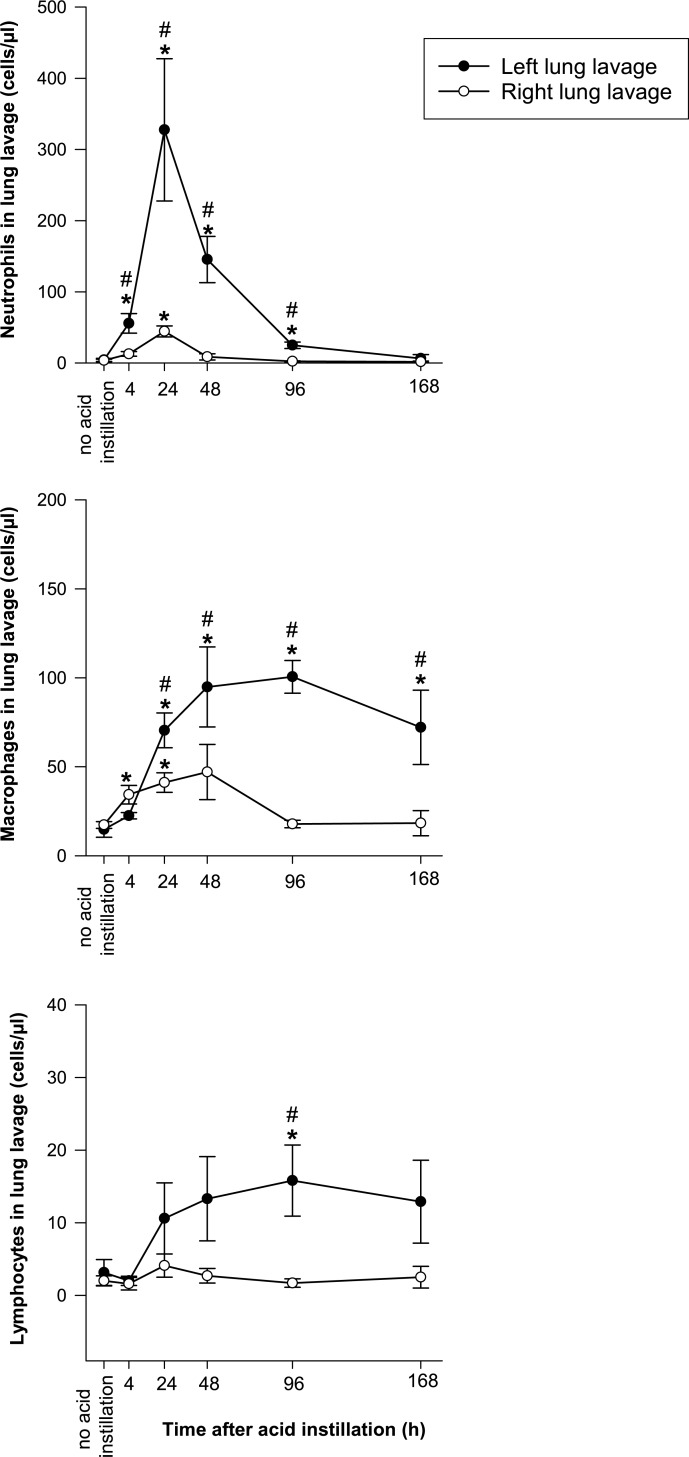
Neutrophil, macrophage and lymphocyte counts in left and right lung lavage fluid following left lung acid instillation. Please note that scales on the y-axes are different. * = p < 0.05 vs. no acid instillation. # = p < 0.05 vs. right lung.

#### Cytokine levels in lung lavage fluid (Fig 3, S3 and S4 Tables)

IL-6 in lung lavage was detected in none of the animals at baseline. After acid injury there was an early increase in IL-6 in lung lavage fluid in both lungs, which was stronger in the left lung, with statistically higher values until 48h after instillation. At 96 h, detectable levels were present in only 1 animal.

**Fig 3:**
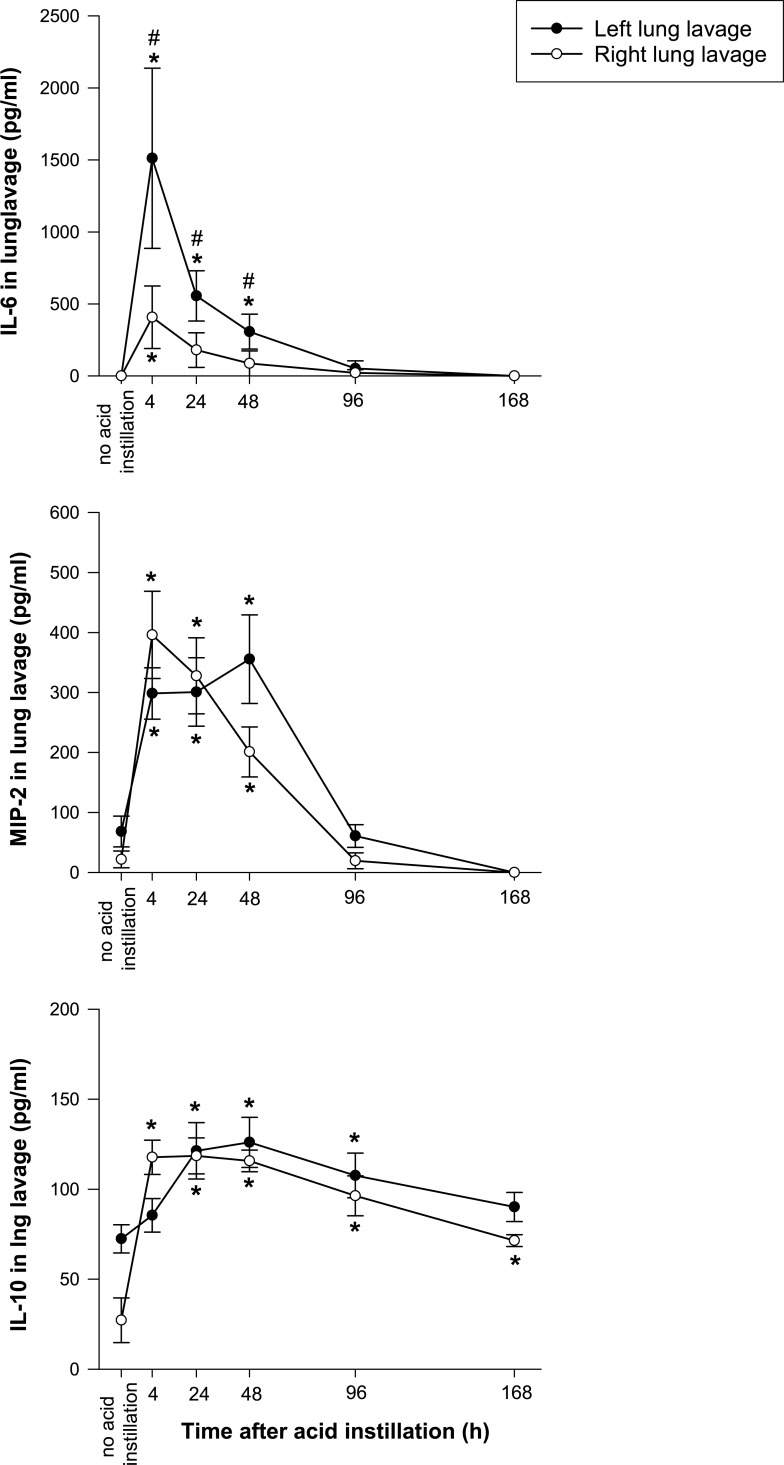
Concentrations of interleukin 6 (IL-6), macrophage inhibitory protein 2 (MIP-2) and interleukin 10 (IL-10) in left and right lung lavage fluid following left lung acid instillation. Please note different scales on the y-axes. * = p < 0.05 vs. no acid instillation. # = p < 0.05 vs. right lung.

Low levels of MIP-2 were detected in lung lavage fluid in 4 of 5 animals at baseline. Following acid instillation there was an early increase in MIP-2, which was comparable in both lungs. At 96 h, levels were not significantly different from baseline values.

IL-10 was detected in lung lavage fluid of all but 3 animals at baseline. After acid injury there was a statistically significant increase in IL-10 in the right lung at four hours. At the time point 24 hours after injury in both lung sides, comparable levels of IL-10 could be detected. This elevation was statistically significant bilaterally from 24 to 96 hours after acid instillation. Seven days after injury, only right lung lavage IL-10 concentration remained significantly above baseline values.

Procollagen III peptide in lung lavage fluid (S3 Table)

P-III-P levels were near or below the detection threshold in all samples.

IL1-β (S4 Table):

IL1-β in lung lavage fluid was detected in none of the animals at baseline, in approximately 50% of animals in both lungs at 4, 24 and 48 h, in 1 animal at 96 h and in no animal at 168 h. At each time point, levels were similar in left and right lung lavage fluid. Because of the low number of detectable values at each time point no statistical analysis was performed.

#### Cytokine levels in plasma (S5 Table)

Plasma levels of IL1-β, IL-10 and MIP-2 were below the detection threshold in all animals at all time points. IL-6 in plasma was below the detection threshold in all but 3 animals at 24 h and in all but 1 animal at 96 h.

#### Lung histology

**Fig 4:**
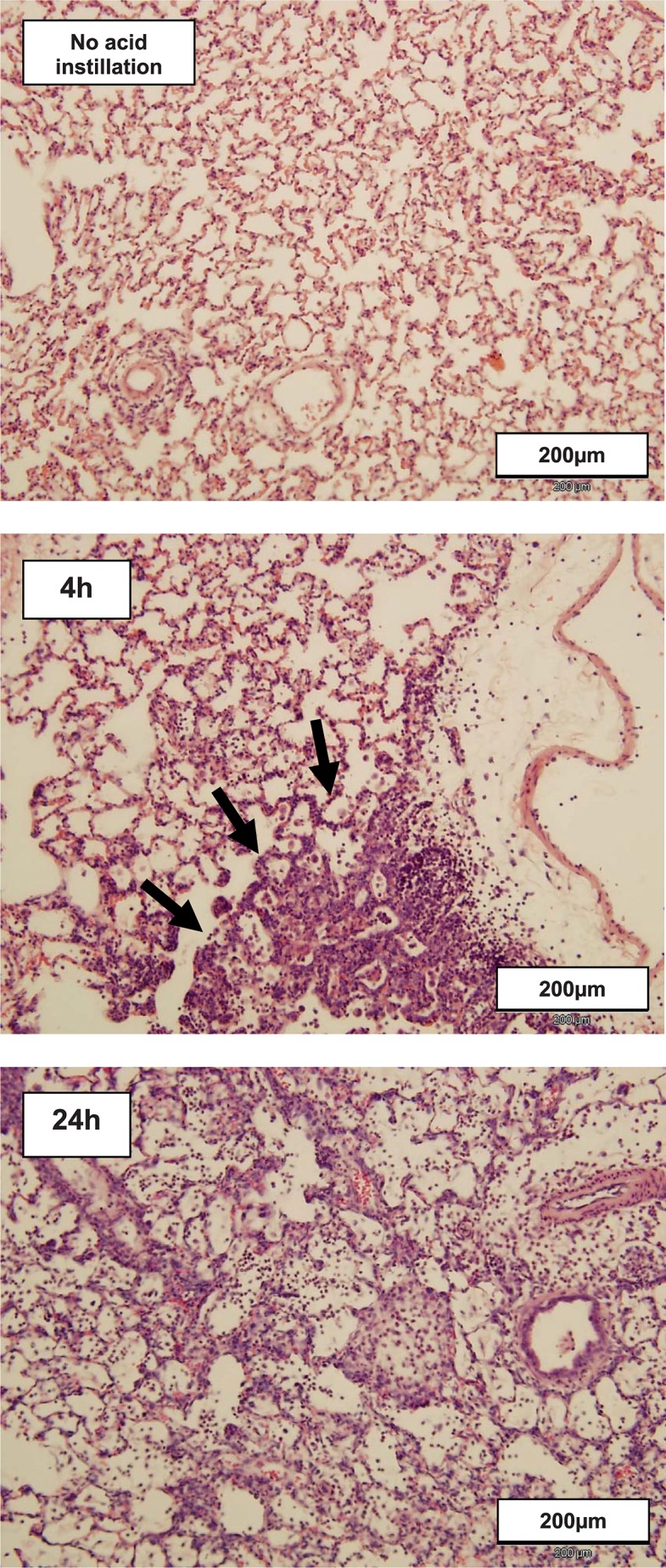
Representative hematoxylin & eosin stained tissue sections from left lungs before and in the early phase after left lung acid instillation. At 4 h after acid instillation focal areas of alveolar inflammation (lower part of tissue section, arrows) were seen adjacent to mostly normal lung tissue (upper part of tissue section). At 24 h after acid instillation a diffuse alveolar and interstitial inflammatory infiltrate, consisting predominantly of neutrophils was the characteristic finding in all animals. Original magnification is x200.

**Fig 5:**
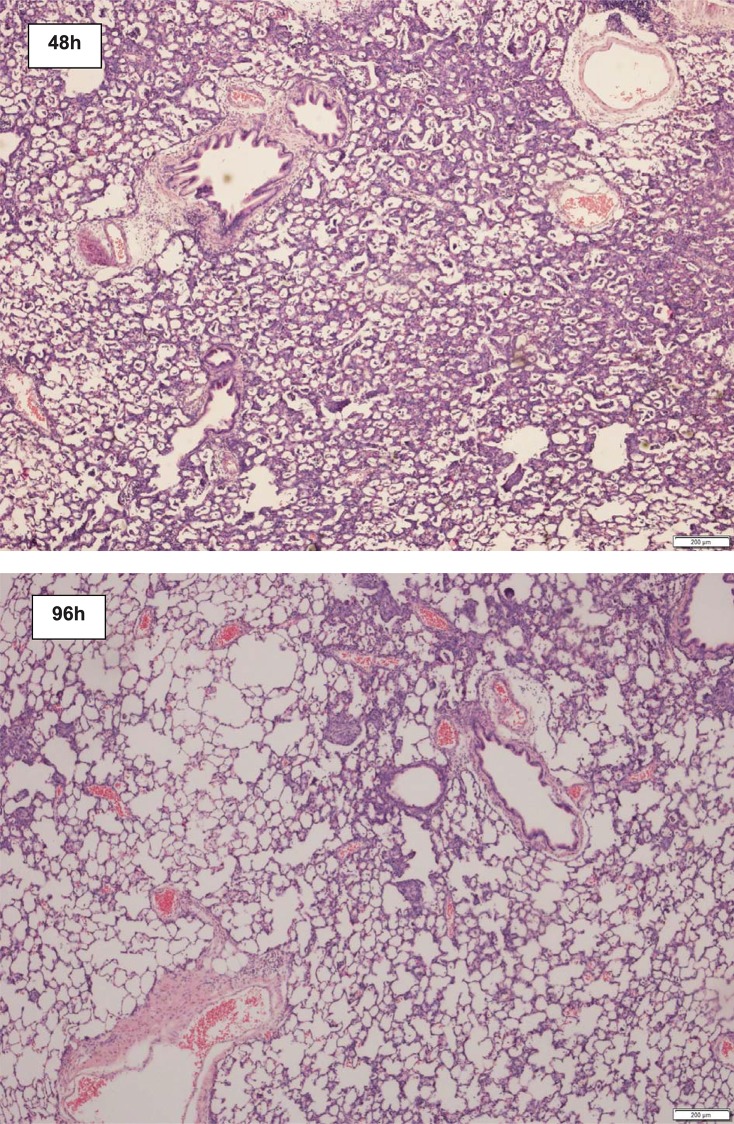
Representative hematoxylin & eosin stained tissue sections from left lungs 48 and 96 hours after left lung acid instillation. At 48h after acid lung-injury, there is widespread cellular infiltration of the lung tissue, predominantly by neutrophil leukocytes. At 96h after acid instillation the interstitial cellular infiltration is beginning to resolve. There are signs of alveolar damage and disruption. Original magnification is x200.

In the early course after left lung acid injury, left lungs revealed marked inflammation, hyaline membranes and, occasionally, alveolar destruction (Fig 4). Between 48 and 96 hours after acid injury, the cellular infiltrate starts to resolve (Fig 5)

**Fig 6:**
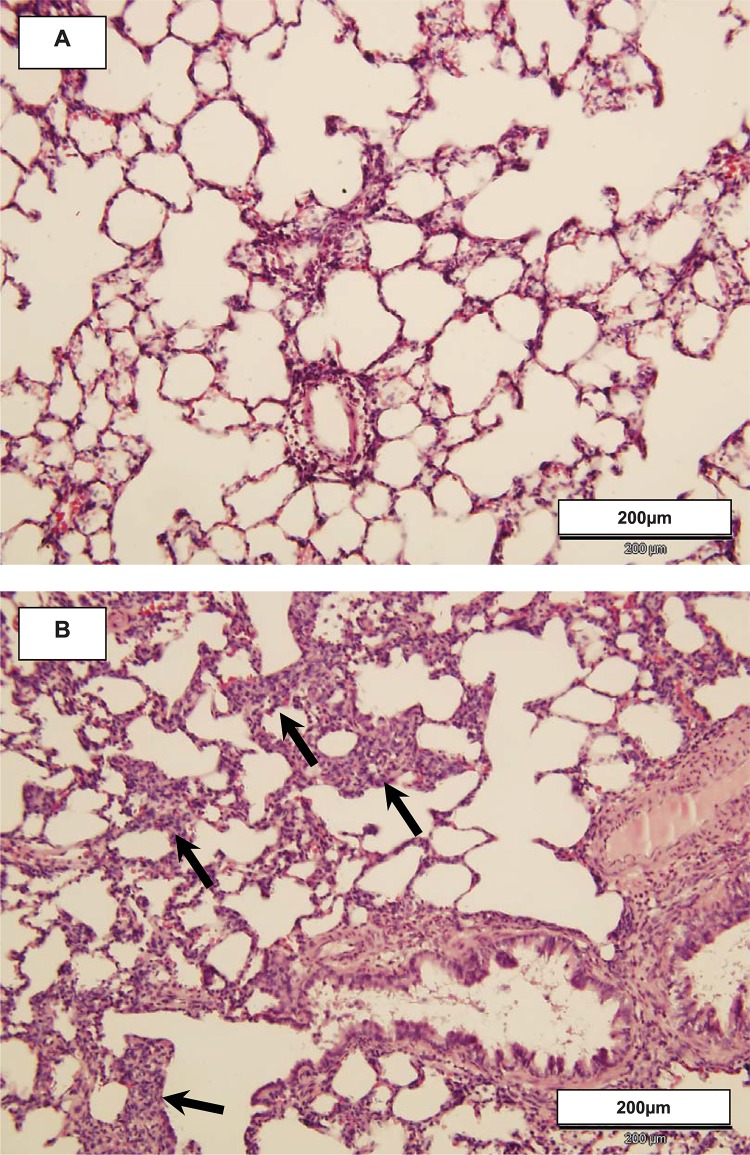
Representative hematoxylin & eosin stained tissue sections from left lungs 7 days after left lung acid instillation. At that time, gross alveolar cellular infiltration was absent and large tissue areas appeared unaffected as shown in section A. However, as shown in section B there were scattered areas with histopathologic alterations characterized by expansion of alveolar walls and destruction of normal alveolar architecture (arrows). Similar findings were present in left lungs of each animal at that time. Original magnification is x200.

Seven days after acid instillation left lungs, while presenting largely without visible alteration, still exhibited focal areas of interstitial cellular infiltration and consolidation (Fig 6) but no increase in staining indicative of fibrosis. There were no significant histopathologic alterations in right lungs.

### Longitudinal experiments

#### Oxygenation (Fig 7)

Compared to baseline, arterial oxygen tension decreased significantly at 4 and 24 h after acid instillation.

**Fig 7:**
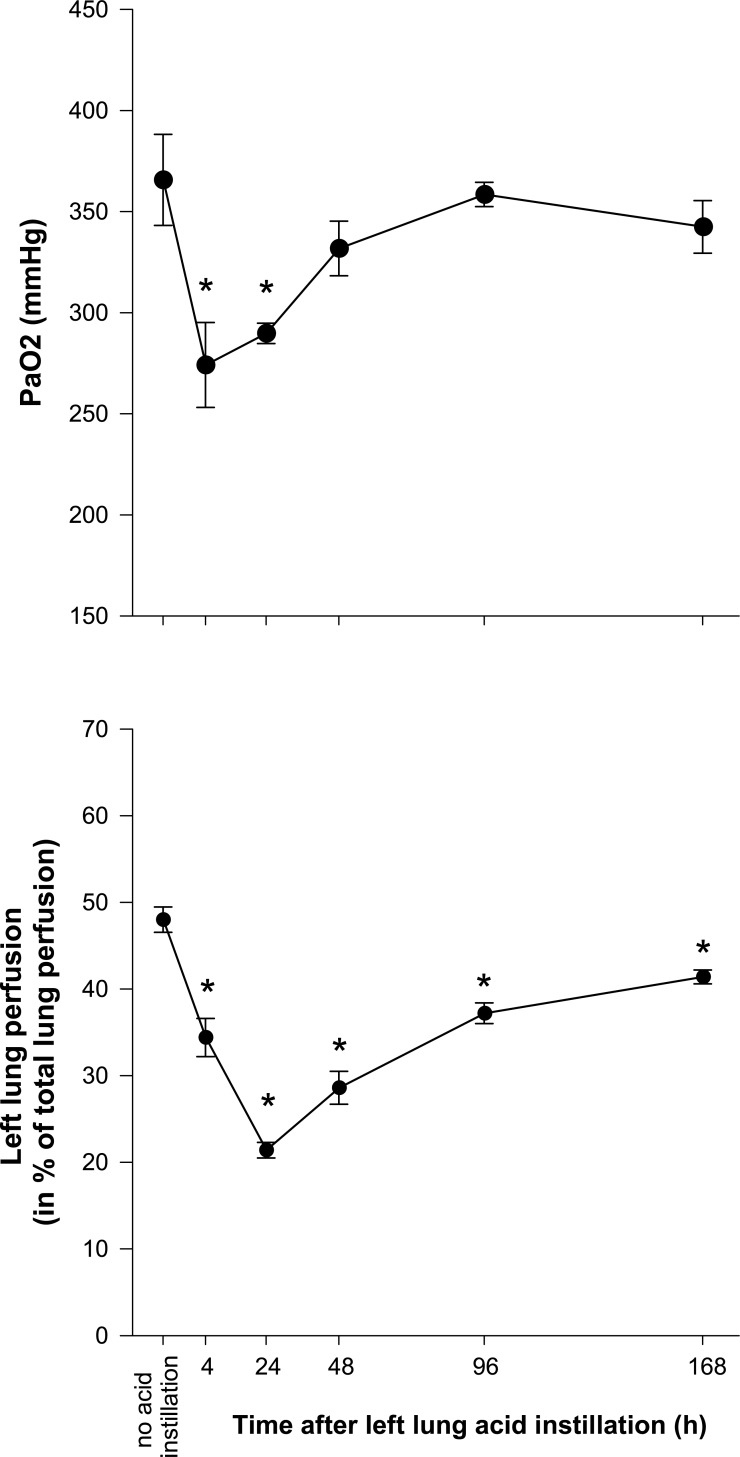
Arterial oxygen tension (PaO2) and left lung perfusion following left lung acid instillation. * = p < 0.05 vs. no acid instillation.

#### Lung perfusion (Fig 7)

Left lung acid injury caused a significant decrease in left lung perfusion. This effect was most pronounced at 24 h and still significant at 168 h after acid instillation.

#### Blood neutrophil and lymphocyte counts (Table 1)

Blood neutrophil counts increased significantly in the early course after left lung acid injury and returned to baseline values after 24 hours. Regarding blood lymphocyte counts there was an early decrease after left lung acid injury with numbers reaching baseline values again at 48 hours.

**Table 1:** Blood leukocyte counts (cells/ μl) prior (= baseline) and after left lung hydrochloric acid injury.

Time point	neutrophils	lymphocytes
baseline	578 ± 123	8177 ± 945
4 hours	4498 ± 890 *	6050 ± 529 *
24 hours	889 ± 103	6094 ± 353 *
48 hours	887 ± 155	7921 ± 1224
96 hours	636 ± 135	8364 ± 914
168 hours	671 ± 130	6503 ± 688

Values are given as mean ± SEM.

* = p < 0.05 vs. baseline.

## Discussion

This study comprehensively characterizes, in a zero-mortality animal model, the response to unilateral lung acid aspiration in both directly injured and non-injured lung areas.

While the early response to experimental lung acid injury has been repeatedly a topic to research, the strength of our study is that it adds to existing knowledge by combining (i) a longer observation period after (ii) focal acid-injury with (iii) a broad array of markers assessed at all pre-defined time points.

Investigating a period of seven days after acid instillation permitted evaluation of early pulmonary injury as well as of the subsequent course of the pro and anti-inflammatory response and potential recovery.

By confining acid instillation to one lung only, our model simulates the finding, that in clinical acute lung injury severely affected and largely preserved lung regions are often present simultaneously [18–20] and it offers the prospects to explore these regions separately. In the early course after focal acid injury, potentially detrimental effects in distant, not directly injured lung areas [21–23] and differential susceptibility of severely and less injured lung areas to further, potentially noxious stimuli have been shown [25]. These findings suggest that evaluating the time course of the response in both areas separately is important.

In the light of some controversy on the role of inflammatory markers in experimental lung injury [28], assessing concomitant functional and morphologic pulmonary alterations facilitates appraisal of inflammatory findings. Consequently, besides pulmonary inflammation, we also evaluated lung edema, histology and arterial oxygenation. In addition, by evaluating selective lung perfusion at each time point, the study gives new information on the time course of redistribution of pulmonary blood flow so far not well described after acid injury.

Regarding cellular markers of inflammation, we differentiated and quantified leucocyte subpopulations (including lymphocytes) in lung lavage thus avoiding a narrow focus on e.g. alveolar neutrophils only, the most intensely looked at cell population following lung injury.

In line with other studies [6, 13, 14, 24] we found a strong early response in the acid instilled lung, characterized by pulmonary edema, cellular infiltration and cytokine expression. While some of the injury markers (e.g. neutrophils, IL-6) in the acid injured lung declined to baseline levels within one week, others, such as alveolar macrophage and lymphocyte counts did not. Of note, an important role of inflammatory cells other than neutrophils (e.g. alveolar macrophages) in lung acid injury has been shown previously [7]. Together with persistent focal histologic alterations, these findings indicate incomplete resolution of direct injury at 7 days after acid instillation. Although exploration of mechanisms of lung repair or defective healing was not the main focus of the study, the findings in lung lavage (procollagen III peptide) and in light microscopy at least are not suggestive of significant fibrosis evolving at 7 days after acid injury. Rather, lung histology at 7 days, revealing focal areas of consolidation and cellular density, is indicative of residual inflammation. Animal models of lung fibrosis, suggest that fibrosis becomes established after post-injury day 14 [29].

In the non-acid instilled lung, no edema and a less severe, time-limited, inflammatory response occurred, with all variables declining to baseline levels within 96 hours. Other studies indicate that propagation of inflammatory effects into the non-instilled lung may occur via the systemic route [30] and point to an important role of circulating neutrophils in mediating inflammation [21, 22]. The increase in blood neutrophil counts seen early after acid injury, suggests that the latter mechanism played a role in our model.

The peak of lung inflammation and edema coincided with functional respiratory impairment as indicated by a transient but pronounced decrease in oxygenation, which in turn was accompanied by progressive hypoperfusion of the acid instilled lung. The latter effect was most pronounced at 24 hours while the nadir in arterial oxygen tension was seen 4 hours after the insult. This suggests that redistribution of lung perfusion away from poorly aerated acid instilled lung tissue towards the opposite lung, most likely due to hypoxic pulmonary vasoconstriction, prevented a sustained decrease in oxygenation. The subsequent normalization of left lung perfusion in conjunction with amelioration of arterial oxygen tension most likely reflects significant repair in acid injured lung areas.

Further data on lung perfusion after focal acid injury are scarce. One study reported hyperperfusion of acid instilled lung areas in the very early period (10 min after injury), but does not provide lung perfusion data on the subsequent course [31]. We speculate that the change from immediate hyperperfusion to early hypoperfusion takes place in the first hours after focal acid injury, a period missed in this as well as in our study.

So far only few rodent studies explored the effect of lung acid injury beyond the first 48 hours, in general (and consistent with our findings) reporting an early inflammatory response and incomplete repair with residual pulmonary alterations at 7, 10 or 21 days, respectively [15–17]. Beyond this general similarity however, comparison of these studies with our experiments is difficult because of methodological differences: In one study bilateral acid injury was induced and mechanical ventilation was employed prior to sampling or measurement procedures [15]. Another study provided data on the response up to 24 hours and at 2 weeks after focal acid injury but did not characterize the period in between [16]. A third study mainly focused on the value of positron emission tomography to characterize the inflammatory response up to 7 seven days while using other markers of lung impairment at 21 days after unilateral acid injury [17].

Hence our experiments add new information to existing knowledge on the response to experimental lung acid injury and to the findings of these studies by providing data on a broad (and at all time-points identical) portfolio of markers. This includes lung perfusion, which was obtained at time points grasping the period from early injury up to one week after acid insult, without skipping the intermediate course (i.e. 48–96 hours).

Our study has some limitations. First, our model represents a moderate pulmonary insult and results may be different in more severely affected animals. However, the dose of hydrochloric acid used is in line with other studies [13, 14] and higher doses bear the risk of significant mortality. Second, experimental acid injury may differ from the clinical situation of aspiration of acidic gastric contents, which may be a mixture of acidic and non-acidic, and particulate and non-particulate material. Third, one could speculate that absence of some indices of lung injury at 7 days indicates failure of the initial insult. However, the fact that elevated alveolar cytokine levels and differences between left and right lung inflammatory indices were present even in that group, suggests that low or normal levels of some other markers of lung injury truly reflect recovery and not failure of the model.

Lastly, our findings regarding alveolar cytokine levels deserve some discussion. Following specific types of lung injury, different kinetics of different cytokines were reported [9, 11]. Thus, the interval between insult and sampling is an important factor regarding the interpretation of such mediators. While discrimination between injured and non-injured lung regions and the course of inflammation may be reflected well by one marker (e.g. IL-6 in our study) other markers may be less helpful in this regard. For example, the peak of pro-inflammatory markers expressed in the immediate course after acid injury (e.g. TNF-α and IL-1β) may be missed, if (as in our study) earliest samples are taken at 4 hours [12, 14]. Our findings support the notion that alveolar cytokine levels alone should only cautiously be used to assess severity of experimental lung injury [32, 33].

Our findings may be relevant in further experimental as well as in clinical settings. Most studies in the field of experimental lung injury focus on the immediate and short-term pulmonary effects. Given the 100% survival rate at 7 days in our model and the fact that, among different types of lung injury, cardiovascular stability has best been maintained after pulmonary acid aspiration [34], focal acid instillation is suitable for investigations of the long-term effects of a pulmonary insult as well as for two-hit scenarios. Two-hit models are valuable tools to investigate e.g. the interaction of lung injury (= first hit) and subsequent mechanical ventilation (= second hit) [35–38]. So far, most such studies applied both insults in a timely closely related fashion. Our data can be a helpful basis for the design of further experiments which may establish longer intervals between lung acid injury and a second intervention (such as mechanical ventilation) and they may facilitate to differentiate effects of the first from those generated by the second intervention.

Mild, subclinical pulmonary inflammation has been shown to increase pulmonary susceptibility to further injurious stimuli, such as mechanical ventilation [35] or increased regional blood flow and perfusion pressure [25]. In this light, both the transient mild response in the right lung, as well as the residual inflammation at 7 days in the left lung in the present study point to increased pulmonary vulnerability.

## Conclusions

This study demonstrates that after unilateral lung acid injury a bilateral pulmonary response is present for approximately 96 hours including profound inflammation and detrimental functional effects in directly damaged and mild to moderate inflammatory effects in not directly injured lung.

At seven days after the insult, resolution was complete in the not directly injured lung areas. Although significant, recovery in the directly injured lung appeared incomplete and persistence of inflammatory and histopathologic alterations in these areas may indicate that vulnerability to further noxious stimuli is still increased after one week.

Regarding clinical practice, where treatment and interventions following pulmonary aspiration may be initiated at variable intervals after the event, our findings may increase the awareness as to how long increased pulmonary vulnerability may be present. Detailed knowledge on evolution and resolution of lung injury following a pulmonary insult may be helpful to improve treatment strategies, e.g. by choosing lung protective ventilator settings.

## Supporting information

S1 Table Dataset animals. (XLSX)

S2 Table Left lung perfusion. (XLSX)

S3 Table Procollagen III peptide (PIIIP). (XLSX)

S4 Table Cytokine levels in lung lavage fluid. (XLSX)

S5 Table Cytokine levels in plasma. (XLSX)

S1 Timeline Experimental groups. (PDF)

S1 Text Checklist ARRIVE guidelines. (PDF)
