# Supplementary material for: Characterization of the seven-day course of pulmonary response following unilateral lung acid injury in rats
Source: PLoS One. 2018 Jun 4;13(6):e0198440. doi: 10.1371/journal.pone.0198440 (PMC5986146; doi:10.1371/journal.pone.0198440)
Supplement: S1 Timeline — (PDF) [file pone.0198440.s006.pdf]

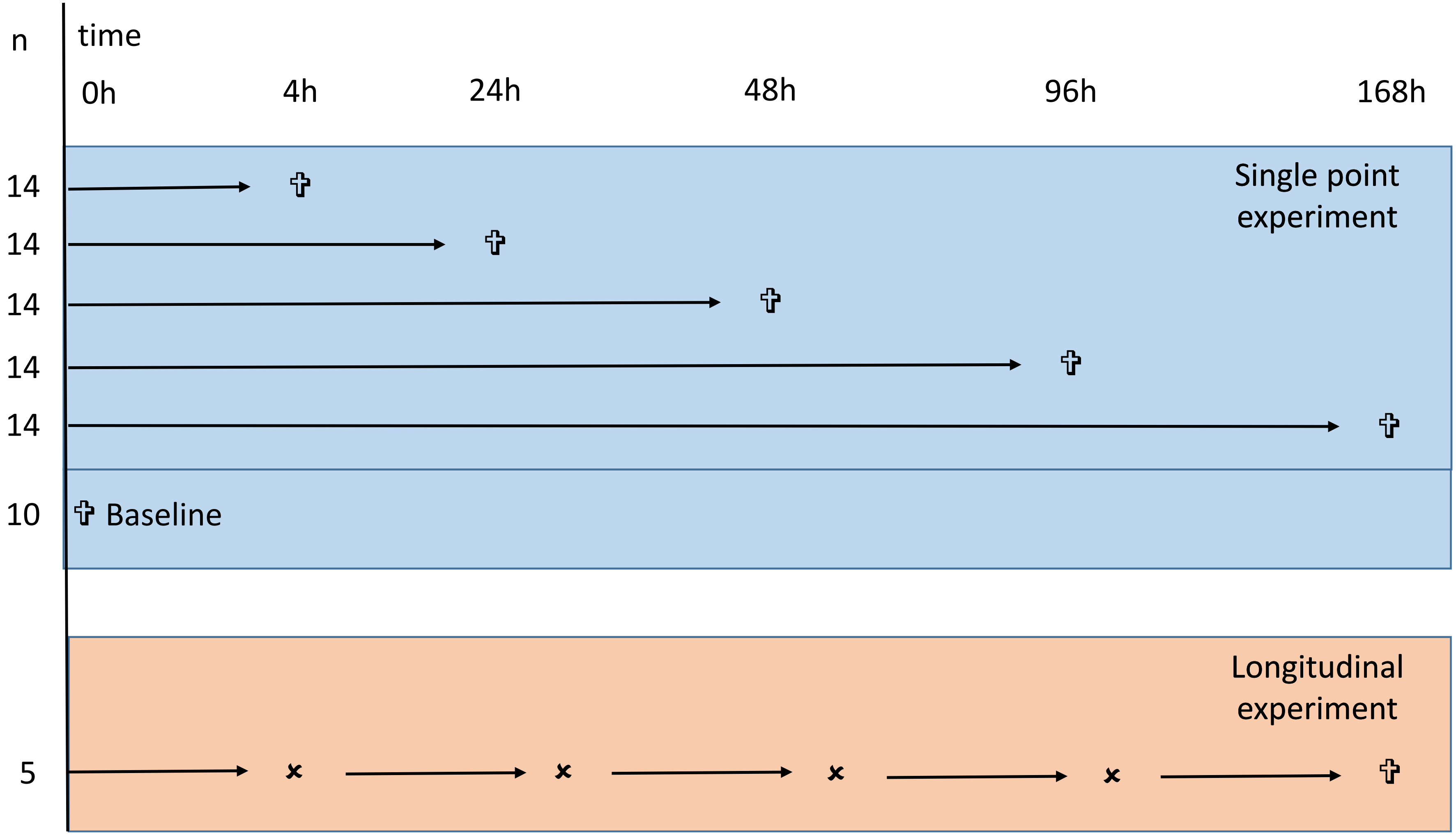

S6 Experimental groups and time points. h = hours after acid injury, ✞ = end point / sacrifice of animal, ✗ = blood sampling, microsphere injection.
